# Effects of digital-based interventions on muscular strength in adults: a systematic review, meta-analysis and meta-regression of randomized controlled trials with quality of evidence assessment

**DOI:** 10.1080/07853890.2023.2230886

**Published:** 2023-07-15

**Authors:** Armin Paravlic, Luka Šlosar, Ensar Abazovic, Uros Marusic

**Affiliations:** a Faculty of Sport, University of Ljubljana, Ljubljana, Slovenia; b Science and Research Centre Koper, Institute for Kinesiology Research, Koper, Slovenia; c Faculty of Sports Studies, Masaryk University, Brno, Czech Republic; d Department of Health Sciences, Alma Mater Europaea – ECM-, Maribor, Slovenia; e Faculty of Sport and Physical Education, University of Sarajevo, Sarajevo, Bosnia and Herzegovina

**Keywords:** (registration number: CRD42022337043), Cognitive training, physical function, strength, neurodegenerative disorders, frailty, older adults

## Abstract

**Background:**

In the last three decades, both medical and sports science professionals have recognized the considerable potential of digital-based interventions (DBI) to enhance the health-related outcomes of their practitioners.

**Objectives:**

This study aimed to investigate the effectiveness and potential moderators of DBI on measures of muscular strength.

**Methods:**

Six databases (PubMed/MEDLINE, Web of Science, SportDiscus, Embase, Cochrane Register of Controlled Trials and Google Scholar) were searched for eligible studies up to June 2022. The GRADE, PEDRO, and TIDieR checklists were used to assess the quality of evidence, methodology, and completeness of intervention descriptions, respectively.

**Results:**

A total of 56 studies were included in the meta-analysis (*n* = 2346), and participants were classified as healthy (*n* = 918), stroke survivors (*n* = 572), diagnosed with other neurological disorders (*n* = 683), and frail (*n* = 173). The DBI showed a small effect (standardized mean difference [SMD] = 0.28, 95% CI 0.21 to 0.31; *p* < 0.001) on strength, regardless of the type of intervention, control group, or tested body part. More specifically, while splitting the studies into different subgroups, a meta-analysis of 19 studies (*n* = 918) showed a small effect (SMD = 0.38, 95% CI 0.12 to 0.63; *p* = 0.003) on strength in the asymptomatic population. Similarly, small but positive effects of DBI were observed for stroke survivors (SMD = 0.34, 95% CI 0.13 to 0.56; *p* = 0.002), patients diagnosed with other neurological disorders (SMD = 0.17, 95% CI 0.03 to 0.32; *p* = 0.021), and the frail population (SMD = 0.25, 95% CI 0.0 to 0.5; *p* = 0.051). Sub-group analysis and meta-regression revealed that neither variable modified the effects of the DBI on measures of strength.

**Conclusions:**

Overall, DBI may serve as an effective method to improve measures of strength in adults, regardless of their health status as well as the type of digital device, the presence of human-computer interaction, and the age of participants. In addition, the DBI was found to be more effective than traditional training or rehabilitation methods.

**KEY MESSAGES**
- Digital-based intervention (DBI) is effective in improving measures of muscular strength in adults regardless of participants’ health status
- DBIs were equally effective for strength improvements in lower and upper limbs
- Although, DBIs were found to be effective in improving muscular strength, most studies did not follow strength training guidelines when prescribing the interventions

## Introduction

Muscular strength is one of the most widely investigated measures of physical performance [1,2]. Among adults, higher levels of muscular strength were shown to be highly correlated with health-related outcomes, lower risk of different chronic non-communicable disease events and comorbidities, reduced risk of falls, longevity, increased physical independence, and quality of life in general [1,3,4]. In addition, for already symptomatic populations diagnosed with musculoskeletal system diseases (e.g. low back pain, osteoporosis, osteoarthritis, etc.), metabolic diseases (e.g. obesity, diabetes), and cardio-cerebral vascular system diseases (e.g. coronary artery disease and stroke survivors), lower levels of muscular strength were found to be a significant predictor of poorer rehabilitation outcomes [3,5–7]. Thus, improving general physical fitness and muscular strength in particular should be a priority when prescribing physical exercise for the elderly population [3].

Although traditional exercise modalities such as resistance training and aerobic exercise improve physical fitness and consequently lead to numerous positive health-related outcomes, some individuals might lose interest in and motivation to perform these traditional exercise modalities for longer periods [8]. Hence, the positive effects of exercise may be absent or even unachievable in the long term.

With the advancement of technology, the gaming sector has seen tremendous growth and widespread adoption, owing to the introduction of cutting-edge, user-friendly gadgets and devices at reasonable rates. Over the last three decades, both medical and sports science professionals have recognized the considerable potential of using these technologies to enhance the health-related outcomes of their clients [9–11]. Through interactions with multimodal environmental and physical stimuli, the use of digital devices in the exercise domain encourages practitioners to engage in physical activity [12]. This may involve the use of motion sensors, altering the environment by incorporating real-world aspects into virtual reality (VR), or incorporating virtual elements into a real-world setting [9–11].

Preliminary evidence suggests that stroke survivors, patients on haemodialysis, following total knee arthroplasty, with spinal cord injury, and cognitive decline benefitted from VR-based interventions to a greater extent compared to conventional rehabilitation [13–17]. A recent review showed that exergaming, a form of digital-based intervention (DBI), showed promising results in enhancing strength outcomes in people with diverse health statuses compared to traditional care [18,19], whereas some original studies found conflicting results [20,21]. For the purposes of this systematic review and meta-analysis, DBI refers to those in which participants interact individually with an immersive or non-immersive digital environment generated by computers, consoles, head-mounted displays, or related devices. The methodology used for the aforementioned DBI varied considerably, as did the participant demographics (such as age, sex, and health status), experimental setting (such as the type of DBI used, its duration, intensity, and complexity), control group, and measures of interest. Inconsistency in defining interventions is another one of the topic’s most important problems, making it quite challenging to come to any firm conclusions about the effectiveness of DBI on measures of interest.

To solve some of the aforementioned issues in the literature, the current article aimed to conduct a systematic review and meta-analysis of studies investigating the effects of DBI on measures of muscular strength. Thus, the purpose was fourfold: (i) to investigate the effects of DBI on measures of muscular strength in general; (ii) to compare the effects of DBI versus non-exercise control groups on changes in muscular strength; (iii) to compare the effects of DBI versus traditional exercise groups on changes in muscular strength; and (iv) to investigate whether the effects of DBI differ between different DBI types (e.g. PC-exergame, PC-no-exergame, VR-exergame vs. VR-no-exergame) or DBI intervention focus (upper vs. lower body parts).

## Materials and methods

### Protocol and registration

This systematic review was performed according to the Preferred Reporting Items for Systematic Reviews and Meta-Analyses guidelines [22]. The protocol was registered in the prospective international register of systematic reviews (PROSPERO; registration number: CRD42022337043).

### Search strategy and study selection

To identify all potentially relevant data from the experimental studies, an initial systematic literature search was conducted in July 2021 by one author (AP). Updated searches were additionally conducted between 10^th^ and 15^th^ February 2021 and 17^th^ June 2022, to include new relevant studies by two authors (LS and EA). Both the initial and updated searches included the following databases: PubMed/MEDLINE, Web of Science, SportDiscus, Embase, Cochrane Register of Controlled Trials, and Google Scholar. Electronic databases were searched using the following keywords or their combination: ‘virtual reality’, ‘augmented reality’, ‘mixed reality’, ‘extended reality’, ‘video games’, ‘kinect’, ‘wii’, ‘exergames’, ‘training’, ‘intervention’, ‘exercise’, ‘strength’, ‘power’, ‘force’, ‘functional performance’, ‘ROM’, ‘effects’, ‘physical function.’ The selected studies also underwent manual reference-list verification and citation monitoring. Two reviewers (LS and EA) independently assessed the study titles and abstracts to determine if they satisfied the eligibility criteria. We included studies recruiting female and/or male adults, regardless of their health status aimed to investigate the effects of DBI intervention. Measures of interest were compared: (i) in general between experimental and control group; (ii) between different types of DBI (PC-exergame vs. PC-no-exergame vs. VR-exergame vs. VR-no-exergame); (iii) between different types of the control group (passive – where no physical and/or cognitive intervention was applied vs. active – where some form of physical and/or cognitive intervention was applied); (iv) DBI focus (upper vs. lower body parts); Main outcome were measures of strength and/or power performance. Also, only studies that were randomized and published in peer-reviewed journals and had a length of less than a week as well as studies that included at least one control group were qualified.

Studies were excluded if they did not have a comparison group, did not meet predetermined inclusion criteria, and those studies from which we could not extract enough information to calculate the effect size and include them in the qualitative data synthesis.

### Data extraction

Data were independently extracted by two reviewers (LS and EA). Consensus or arbitration by a third reviewer (AP) was used to settle any disputes between the reviewers [23]. The following information was extracted from the study: a) study characteristics, such as the author(s), title, and year of publication; b) participant information c) description of the intervention, such as specific types of DBI used, duration, intensity, and weekly frequency; and d) study outcomes, such as measures related to strength- and power.

Using the PEDro scale, the listed studies’ methodological quality was evaluated separately by two reviewers (LS and EA) [24]. The PEDro scale consists of 11 items designed to assess methodological quality [24]. The Grading of Recommendations Assessment, Development and Evaluation (GRADE) approach was used to evaluate the quality of the evidence, and categories were formed as previously recommended [25]. Also, the experimental and control groups’ intervention descriptions were evaluated for completeness using the Template for Intervention Description and Replication (TIDieR) checklist [26].

### Statistical analysis

Meta-analyses were performed using the Comprehensive Meta-analysis software (version 3.0; Biostat Inc., Englewood, NJ, USA). For all the reported outcome measures, standardized mean differences (SMD) with 95% CIs were calculated. Due to differences in the outcomes assessed and measurement scales used between studies, general strength and power assessments were pooled [27]. A random-effects model was used for all comparisons. In addition, a sensitivity analysis was performed using both fixed- and random-effects models and by removing one study from the analysis.

Furthermore, a random-effects meta-regression was performed to examine whether the effects of the DBI on strength performance, in general, were moderated by different training-related variables and participants’ intrinsic characteristics. Training variables were categorized as follows: duration of intervention, weekly frequency, number of training sessions, and duration of a single training session. For participants’ intrinsic characteristics, the female-to-male ratio and age of the participants were considered.

Using Egger’s test to look at the asymmetry of the funnel plots, publication bias was evaluated, and a substantial publication bias was regarded when the p-value was less than 0.10. The following categories were used to categorize the size of the intervention effects on strength performance: trivial (<0.20), small (0.21–0.60), moderate (0.61–1.20), large (1.21–2.00), very large (2.01–4.00), and extremely large (>4.00) [28]. Between-study heterogeneity was investigated using the I^2^ statistic, where values of 25%, 50%, and 75%, respectively, signified low, moderate, and high statistical heterogeneity [29]. The cut-off for statistical significance was set at *p* < 0.05 [28].

## Results

The Egger’s test was performed to provide statistical evidence of funnel plot asymmetry. The results indicated publication bias for meta-analysis summarizing results for the asymptomatic population only (*p* = 0.025). In contrast, no publication bias was found for any other meta-analysis as follows: general strength performance including all studies (*p* = 0.419) and stroke survivors (*p* = 0.801), patients diagnosed with other neurological disorders (*p* = 0.495), and frail subjects (*p* = 0.427).

### Study selection and characteristics

**Figure 1.**
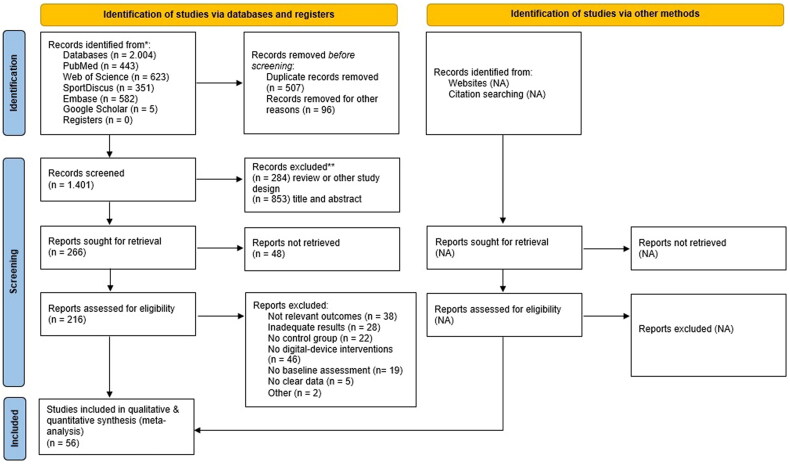
Flow diagram of the study selection process.

The initial search identified 2,004 records, which were reduced to 216 after duplicate removal and title and abstract screening (Figure 1). In the final phase, full-text screening of the remaining articles was performed based on the inclusion/exclusion criteria, resulting in 56 unique records. To summarize the effects relevant to different disorders, the included studies were further grouped based on participant health status and classified as asymptomatic population (*N* = 918), stroke survivors (*N* = 572), other neurological disorders (*N* = 683), and frail population (*N* = 173). Following the recent recommendations for classification of technologies in movement-related research [30], we included one VR-exergame, one VR-no-exergame, 45 PC-exergames, and nine PC-no-exergame studies. Nintendo Wii and Xbox Kinect are the primary digital devices used in PC exergame studies. Sixteen interventions were conducted with non-commercial devices, most of which were stroke survivors. The only VR interventions [18,31] were for other neurological patients, and were all performed with an HTC Vive VR headset. Non-exergame interventions were not identified in the frail population. The detailed characteristics of all included articles are provided in Supplementary Table 1.

### Quality and completeness of reporting

Overall, the included studies were of low to high quality, with PEDro scores ranging from 3 to 9 (out of 10), with an average score of 5.8 ± 1.4 (Table 1).

**Table 1.** Grades of recommendation, Assessment, development and Evaluation (GRADE).

Outcome (muscular strength)	Trials (n)	Participants (n)	SMD	95% LLCI	95% ULCI	I^2^	PEDRO score	Quality of evidence (GRADE)
All subjects	55	2346	0.28	0.17	0.39	77	6	moderate quality
Healthy	19	918	0.38	0.12	0.63	85	6	moderate quality
Stroke survivors	12	572	0.34	0.13	0.56	81	6	moderate quality
Neurological disorders	19	683	0.17	0.03	0.32	56	6	moderate quality
Frail population	5	173	0.25	0	0.5	71	5	very low quality
SMD – standardized mean difference; LCI – lower limits of confidence interval; UCI – upper limits of confidence interval								

**Figure 2.**
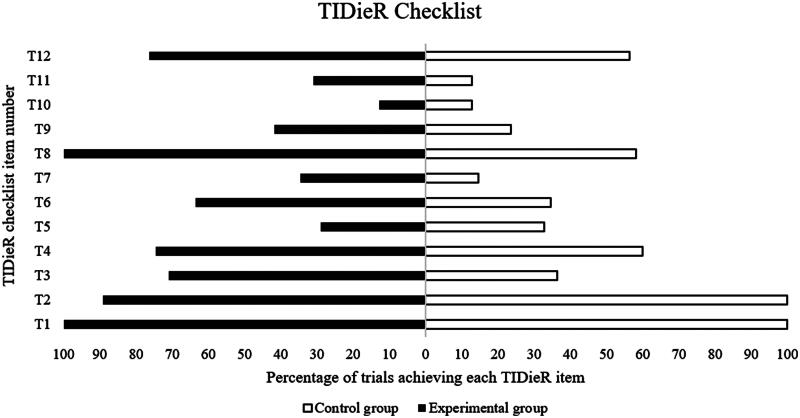
Percentage of studies achieving each Template for Intervention Description and Replication item of the experimental and control groups.

The completeness of intervention reporting was higher for the experimental conditions (mean:60%; range: 13–100%) than for the control groups (mean:45%; range: 13–100%) (Figure 2).

### Effects of digital-based interventions on strength performance in general

#### Summarized effect of all included studies regardless of participants’ health status

A meta-analysis of 56 studies with 2346 participants showed a small effect (SMD = 0.28, 95% CI 0.21 to 0.31; *p* < 0.001) on strength performance (Table 2). The evidence was downgraded from high to moderate owing to moderate to high heterogeneity (I^2^ = 77%; *p* < 0.001) (Table 2). Due to substantial heterogeneity, several subgroup and meta-regression analyses were performed to investigate potential moderators of the observed effect. When the type of intervention (*Q* = 1.62; *p* = 0.654), type of control group (*Q* = 2.35; *p* = 0.125), or tested body parts (*Q* = 0.07; *p* = 0.784) was considered, there were no significant differences between the subgroups (Table 2). Moreover, regression analysis showed that neither variable was a significant predictor of strength performance improvements following the digital-based intervention (Table 3).

**Table 2.** Effects of digital-based interventions on measures of muscular strength in general considering different grouping variables.

Independent variables	SMD	SE	95 % CI		*Z value*	*P value*	*I^2^*(%)	*No. s*	*Q value and (p) between groups*
Cumulated effect (all included studies)									
Fixed effects	0.26	0.03	0.21	0.31	10.08	***p* < 0.001**			
Random effects	0.28	0.05	0.17	0.39	5.10	***p* < 0.001**	77	162	NA
Cumulated effect (all included studies) - Type of intervention									
PC-exergame	0.24	0.06	0.12	0.36	3.88	***p* < 0.001**	81	123	1.62 (0.654)
PC-no-exergame	0.42	0.14	0.15	0.69	3.04	**0.002**	17	28	
VR-exergame	0.47	0.19	0.09	0.85	2.42	**0.016**	0	2	
VR-no-exergame	0.34	0.14	0.06	0.61	2.40	**0.017**	0	9	
Cumulated effect (all included studies) - Type of control group									
Passive group	0.40	0.10	0.21	0.59	4.16	***p* < 0.001**	74	52	2.35 (0.125)
Active group	0.22	0.07	0.09	0.35	3.29	**0.001**	78	110	
Cumulated effect (all included studies) - Body Part									
Upper body	0.26	0.09	0.09	0.43	3.06	**0.002**	71.00	57	0.07 (0.784)
Lower body	0.29	0.07	0.15	0.43	4.06	***p* < 0.001**	91.00	83	
Asymptomatic subjects									
Fixed effects	0.25	0.05	0.15	0.34	5.08	***p* < 0.001**			
Random effects	0.38	0.13	0.12	0.63	2.92	**0.003**	85	38	NA
Type of intervention									
PC-exergame	0.35	0.14	0.08	0.62	2.53	**0.011**	87	33	
PC-no-exergame	0.58	0.37	−0.15	1.30	1.56	**0.120**	0	5	0.32 (0.570)
Type of control group									
Passive group	0.39	0.15	0.10	0.68	2.63	**0.008**	80	30	
Active group	0.33	0.28	−0.22	0.89	1.18	**0.238**	93	8	0.03 (0.858)
Body Part									
Upper body	0.28	0.29	−0.29	0.84	0.96	**0.337**	74	8	
Lower body	0.41	0.15	0.12	0.70	2.74	**0.006**	87	30	0.16 (0.686)
Stroke survivors									
Fixed effects	0.36	0.05	0.27	0.45	7.77	***p* < 0.001**			
Random effects	0.34	0.11	0.13	0.56	3.08	**0.002**	81	44	NA
Type of intervention									
PC-exergame	0.31	0.15	0.02	0.60	2.10	**0.036**	88	26	
PC-no-exergame	0.40	0.18	0.05	0.74	2.24	**0.025**	27	20	0.14 (0.706)
Type of control group - NA only 1 study for group 1									
Body Part									
Upper body	0.37	0.15	0.08	0.65	2.52	0.012	68	29	
Lower body	0.31	0.18	−0.05	0.66	1.71	0.087	89	17	0.07 (0.793)
Neurodegenerative diseases patients									
Fixed effects	0.18	0.05	0.09	0.27	3.67	***p* < 0.001**			
Random effects	0.17	0.07	0.03	0.32	2.30	**0.021**	56	59	NA
Type of intervention									
PC-exergame	0.12	0.09	−0.04	0.29	1.44	0.149	64	45	
PC-no-exergame	0.32	0.28	−0.23	0.88	1.13	0.258	0	3	
VR-exergame	0.47	0.19	0.09	0.85	2.42	**0.016**	0	2	
VR-no-exergame	0.34	0.14	0.06	0.61	2.40	**0.017**	0	9	1.99 (0.574)
Type of control group									
Passive group	0.38	0.14	0.11	0.66	2.76	**0.006**	67	16	
Active group	0.09	0.09	−0.08	0.27	1.07	0.285	50	43	**3.09 (0.079)**
Body Part									
Upper body	0.21	0.11	0.00	0.42	2.00	0.046	27	30	
Lower body	0.13	0.10	−0.07	0.34	1.27	0.204	69	29	0.29 (0.590)
Frail subjects									
Fixed effects	0.21	0.07	0.08	0.34	3.21	0.001			
Random effects	0.25	0.13	0.00	0.50	1.95	0.051	71	19	NA
Type of intervention – *not applicable because only one type of intervention was used among all studies*									
Type of control group									
Passive group	0.47	0.26	−0.05	0.99	1.78	0.075	0	5	
Active group	0.18	0.15	−0.11	0.47	1.21	0.226	77	14	0.92 (0.337)
Body Part									
Upper body	0.00	0.30	−0.60	0.60	0.00	1.000	7	4	
Lower body	0.31	0.15	0.02	0.59	2.12	0.034	76	15	0.83 (0.362)

**Table 3.** Meta regression for training-related variables and participants intrinsic characteristics to predict digital-based interventions effect on measures of muscular strength in general.

	Coefficient	Standard error	95 % lower CI	95 % upper CI	Z value	*p* value
Duration of intervention (weeks)	0.031	0.024	−0.016	0.079	1.290	0.196
Weekly frequency (times per week)	0.080	0.047	−0.012	0.172	1.700	0.089
Number of training sessions during whole study	−0.012	0.007	−0.026	0.002	−1.630	0.103
Duration of single training session	0.000	0.004	−0.007	0.007	0.020	0.984
Females to male ratio	−0.002	0.003	−0.008	0.004	−0.690	0.491
Age of participants (years)	0.006	0.004	−0.002	0.014	1.480	0.139

#### Asymptomatic population

A meta-analysis of 19 studies with a total of 918 participants showed a small effect (SMD = 0.38, 95% CI 0.12 to 0.63; *p* = 0.003) on strength performance (Table 2). The evidence was downgraded from high to moderate owing to high heterogeneity (I^2^ = 85%; *p* < 0.001). Owing to the substantial heterogeneity, several subgroup analyses were performed. When the type of intervention was considered, sub-group analysis showed a small effect following both the PC-exergame (SMD = 0.35, 95% CI 0.08 to 0.68; *p* = 0.011) and PC-no-exergame (SMD = 0.58, 95% CI −0.15, 1.30; *p* = 0.120). Although the effect slightly favoured PC-no-exergame intervention, there was no significant difference (*Q* = 0.32, *p* = 0.570). Similarly, when comparing passive (SMD = 0.39, 95% CI 0.10 to 0.68; *p* = 0.008) and active (SMD = 0.33, 95% CI −0.22, 0.89; *p* = 0.238) controls, there was only a small effect of intervention on strength performance, without significant differences between groups (*Q* = 0.03, *p* = 0.858). Finally, when the effect of the intervention was compared between the upper- and lower-body-focused exercises, a small effect was observed for both groups without statistically significant differences (*Q* = 0.16, *p* = 0.686).

#### Stroke survivors

A meta-analysis of 12 studies with 572 participants showed a small effect (SMD = 0.34, 95% CI 0.13 to 0.56; *p* = 0.002) on strength performance (Table 2). The evidence was downgraded from high to moderate owing to high heterogeneity (I^2^ = 81%; *p* < 0.001). Owing to the substantial heterogeneity, several subgroup analyses were performed. When the type of intervention was considered, sub-group analysis showed a small effect following both the PC-exergame (SMD = 0.31, 95% CI 0.02 to 0.60; *p* = 0.036) and PC-no-exergame (SMD = 0.40, 95% CI 0.05 to 0.74; *p* = 0.025). Although the effect slightly favoured PC-no-exergame intervention, there was no significant difference (*Q* = 0.14, *p* = 0.706). When the effect of the intervention was compared between the upper- and lower-body-focused exercises, a small effect was observed for both groups without statistically significant differences between them (*Q* = 0.07, *p* = 0.793).

#### Other neurological disorders

A meta-analysis of 19 studies with a total of 683 participants showed a trivial effect (SMD = 0.17, 95% CI 0.03 to 0.32; *p* = 0.021) on strength performance (Table 2). The evidence was downgraded from high to moderate owing to moderate heterogeneity (I^2^ = 56%; *p* < 0.001). Owing to the substantial heterogeneity, several subgroup analyses were performed. When type of intervention was considered, sub-group analysis showed trivial (PC-exergame; SMD = 0.12, 95% CI −0.04 to 0.29; *p* = 0.149) to small effects following PC-no exergame (SMD = 0.32, 95% CI −0.23 to 0.88; *p* = 0.258); VR-exergame (SMD = 0.47, 95% CI 0.09 to 0.85; *p* = 0.016) and VR-no-exergame (SMD = 0.34, 95% CI 0.06 to 0.61; *p* = 0.017) respectively. Although the effect slightly favoured the VR exergame intervention, there was no significant difference between the interventions (*Q* = 1.99, *p* = 0.574). Similarly, when comparing passive (SMD = 0.38, 95% CI 0.11 to 0.66; *p* = 0.006) and active (SMD = 0.09, 95% CI −0.08 to 0.27; *p* = 0.285) controls, there was a trivial to small effect of the intervention on strength performance, without significant differences between groups (*Q* = 3.09, *p* = 0.079). Finally, when the effect of the intervention was compared between the upper- and lower-body-focused exercises, a small effect was observed for both groups without statistically significant differences between them (*Q* = 0.29, *p* = 0.590).

#### Frail population

A meta-analysis of six studies with a total of 173 participants showed a small effect (SMD = 0.25, 95% CI 0.0 to 0.5; *p* = 0.051) on strength performance (Table 2). The evidence was downgraded from high quality to very low quality due to moderate to high heterogeneity (I^2^ = 71%; *p* < 0.001), a PEDro score of 5, and imprecision based on small sample size (173 subjects). Owing to the substantial heterogeneity, several subgroup analyses were performed. When comparing passive (SMD = 0.47, 95% CI −0.05 to 0.99; *p* = 0.075) and active (SMD = 0.18, 95% CI −0.11, 0.47; *p* = 0.226) controls, there was only a small effect of the intervention on strength performance, without significant differences between groups (*Q* = 0.92, *p* = 0.337). Finally, when the effect of the intervention was compared between upper- and lower-body-focused exercises, trivial and small effects were observed without statistically significant differences between them (*Q* = 0.83, *p* = 0.362).

## Discussion

The present systematic review and meta-analysis aimed to evaluate the effects of DBI on muscular strength in adults, generalizing across different groups and examining several subgroups of healthy and diseased individuals. As a primary outcome, we found improvements (reflected in small effect size) in strength outcomes independent of health status. Improvements in strength remained small, even when the effect was examined in healthy adults, stroke survivors, and frail individuals. A significant but trivial effect was found in the other neurological disease group, in which the majority of the 683 participants (*N* = 158) had Parkinson’s disease. Thus, our results provide evidence that DBI can improve strength in diverse adult populations and highlight the potential therapeutic benefits of such training.

Using the classification of exergame vs. non-exergame interventions by Šlosar et al. [30], no differences were found between the two groups in any of the population categories, except for the frail population group, in which all interventions were classified as exergames. According to Šlosar et al. [30], the main factors used to classify an intervention as an exergame are i) the presence of a digital device, ii) user-device interaction, and iii) energy expenditure set as higher than 1.5 MET. In our review, we found that energy expenditure was the main factor that determined study allocation. Thus, our results suggest that the dynamic aspect of DBI does not have major effects on strength development in healthy individuals, stroke patients, and other neurological patients. However, the training intensity observed in the selected studies was too low and did not reach the recommended level to induce major increases in strength performance [32].

Regarding the improvement in strength based on body segments trained, although there were no significant differences between the upper and lower body, the effect of the intervention tended to be greater when the upper body was trained. Interestingly, the effect of the intervention did not differ between the passive and active control groups, although there was a tendency for a greater response when the experimental group was compared to the passive control group. For the group of patients with neurological disorders, it was possible to examine the differences among all four interventions considered (PC-exergame, PC-no-exergame, VR-exergame, and VR-no-exergame). The VR exergame proved to be the most effective, whereas PC interventions did not produce any improvements. However, our results may not accurately reflect the capabilities of VR devices because no VR interventions (as defined in Šlosar et al. [30]) were performed on asymptomatic subjects, frail individuals, or stroke survivors. Therefore, future research on larger populations is warranted.

The direct effect of DBI interventions on strength outcome improvement is questionable and requires further investigation, as only [33–35] of the fifty-five included studies were strength-specific. Although intensity and volume have been reported, no reference has been made to the specific strength guidelines for intensity, volume, and periodization. The true performance of such devices cannot be assessed without following physical activity guidelines for strength development (e.g. ACSM guidelines for strength training) that target a specific muscle group. If the main goal of the prescribed training program is to maximize a person’s strength development, there are generally some basic principles that must be followed to obtain the most benefit from the intervention. These principles include training specificity, overload, reversibility, progression, individualization, and periodization [36]. In addition, these training principles should be well organized and periodized by manipulating many strength-training variables, of which training volume and intensity have been shown to be the most important when maximal strength development is the primary goal in older adults.

### Limitations and future directions

This systematic review and meta-analysis have limitations that must be noted. In our meta-analytic calculations, we summarized various strength measurement protocols, ranging from isolated measurements of maximal knee extensor strength to clinical and field tests that assess an individual’s strength endurance and general functioning, in addition to strength performance.

Since our primary focus is on strength outcomes, the authors did not investigate specific health-related outcomes, such as depressive symptoms [37,38] or mental health [39]. Future studies should consider adopting a more holistic approach, encompassing the analysis of health-related data that could potentially exert direct or indirect influence on strength outcomes.

Because of the current lack of scientific evidence, future studies should also examine the effects of DBI interventions in orthopaedic populations with the most common problems, such as anterior cruciate ligament rupture, ankle sprains, or in the rehabilitation of patients with osteoarthritis after joint replacement surgery. The original intention was to summarize the evidence on extended reality (XR) technologies and to investigate the effectiveness of the different technologies by comparing different levels of users’ immersiveness [40]. However, this was not possible owing to the current lack of studies.

Comparing conventional rehabilitation methods with innovative technologies that facilitate rehabilitation and provide telemedicine capabilities will contribute to the development of new (tele)rehabilitation tools that can be used both in medical facilities (e.g. hospitals and rehabilitation centers) and in the home environment [9]. Finally, the observed strength improvements (with a small effect size) could be confirmed without a clear underlying mechanism. Currently, emerging technologies, typically evaluated using the Mobile Brain/Body Imaging (MoBI) approach [41] will provide a link to central nervous system adaptation following acute or chronic exercise or rehabilitation in combination with XR technologies.

## Conclusions

Overall, we found beneficial effects of DBI interventions regardless of the type of digital device and the presence of human-computer interaction. In addition, DBI interventions were more effective than traditional training or rehabilitation methods. Our findings highlight the need for further research on the effects of DBI interventions in the elderly population. Because of the general lack of evidence and the heterogeneity of study designs, our study reports strength improvements, but they remain non-specific. High-quality, well-controlled studies are needed to uncover the underlying mechanisms of strength improvement to promote future use and improvement of DBI.

## Supplementary Material

Supplemental Material

Supplemental Material

## Data Availability

Data related to this manuscript will be provided based on reasonable request to corresponding author.
