# Supplementary material for: Effects of digital-based interventions on muscular strength in adults: a systematic review, meta-analysis and meta-regression of randomized controlled trials with quality of evidence assessment
Source: Ann Med. 2023 Jul 15;55(1):2230886. doi: 10.1080/07853890.2023.2230886 (PMC10901531; doi:10.1080/07853890.2023.2230886)
Supplement: Supplemental Material [file IANN_A_2230886_SM1491.pdf]

Funnel plots and egger's test results

1. All patients

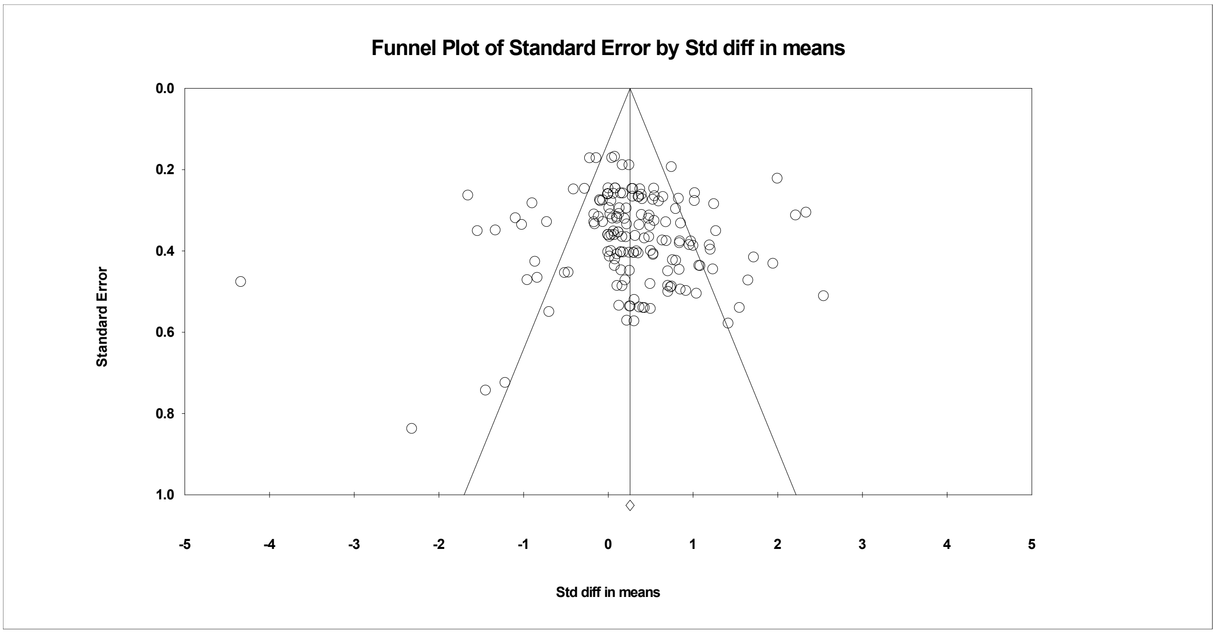

Egger's regression intercept

Intercept	0.45280
Standard error	0.55905
95% lower limit (2-tailed)	-0.65128
95% upper limit (2-tailed)	1.55688
t-value	0.80994
df	160.00000
P-value (1-tailed)	0.20959
P-value (2-tailed)	0.41918

2. Asymptomatic population only

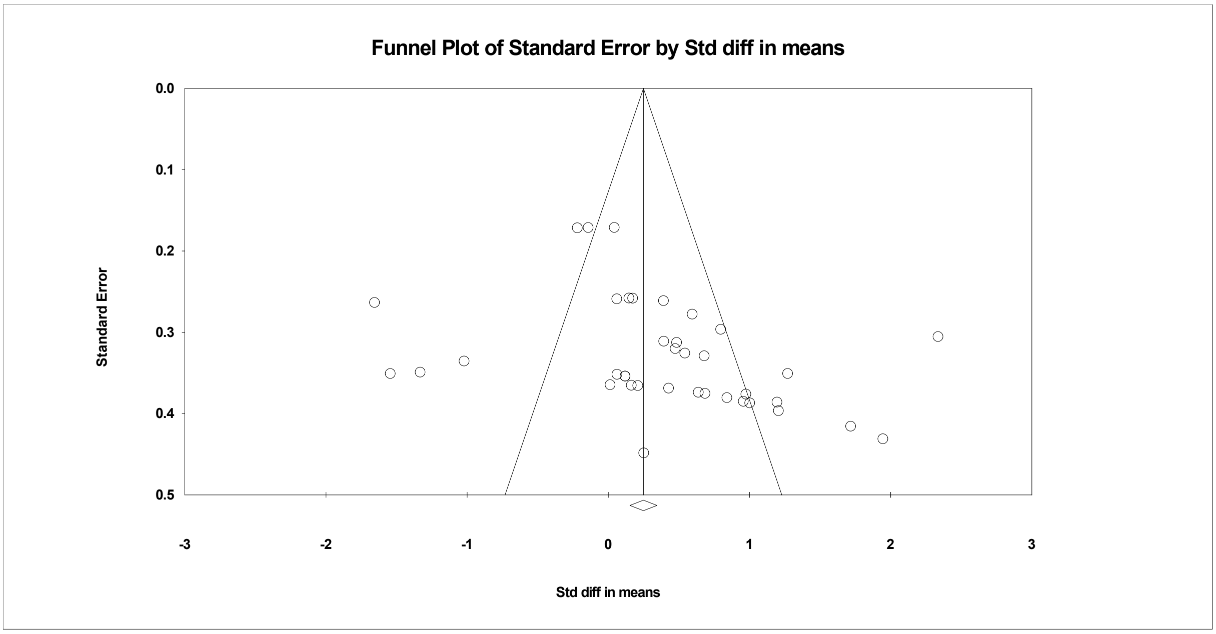

Egger's regression intercept

Intercept	3.58422
Standard error	1.47893
95% lower limit (2-tailed)	0.58481
95% upper limit (2-tailed)	6.58362
t-value	2.42353
df	36.00000
P-value (1-tailed)	0.01026
P-value (2-tailed)	0.02052

3. Stroke survivors

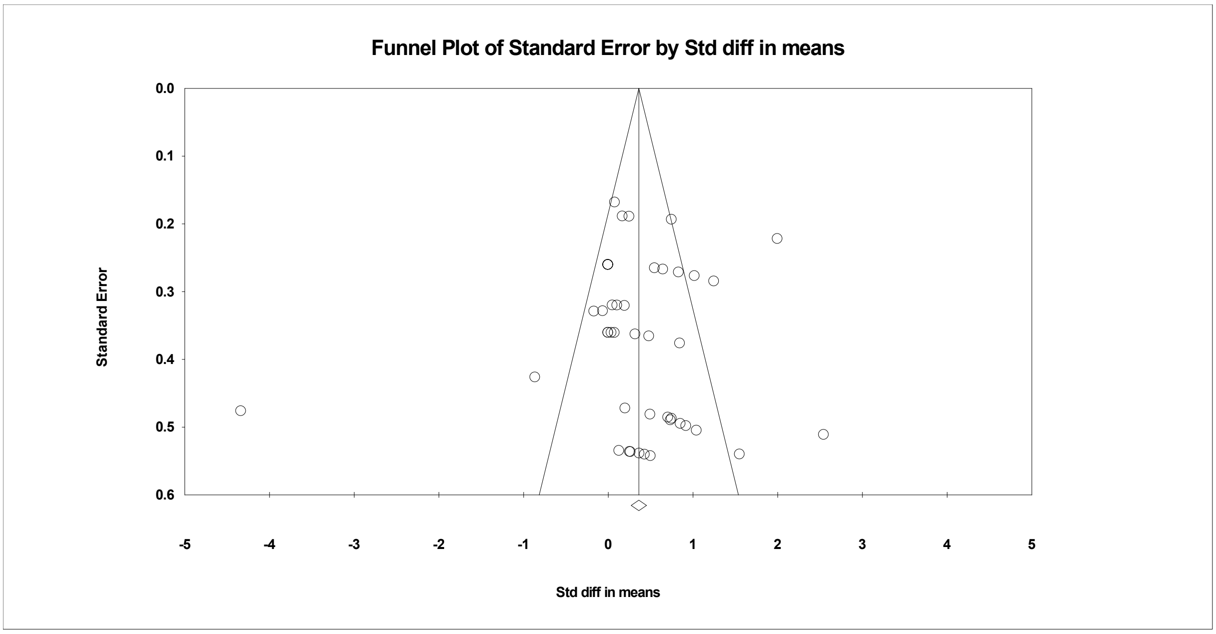

Egger's regression intercept

Intercept	-0.26124
Standard error	1.03201
95% lower limit (2-tailed)	-2.34111
95% upper limit (2-tailed)	1.81864
t-value	0.25313
df	44.00000
P-value (1-tailed)	0.40067
P-value (2-tailed)	0.80134

4. Neurodegenerative diseases patients

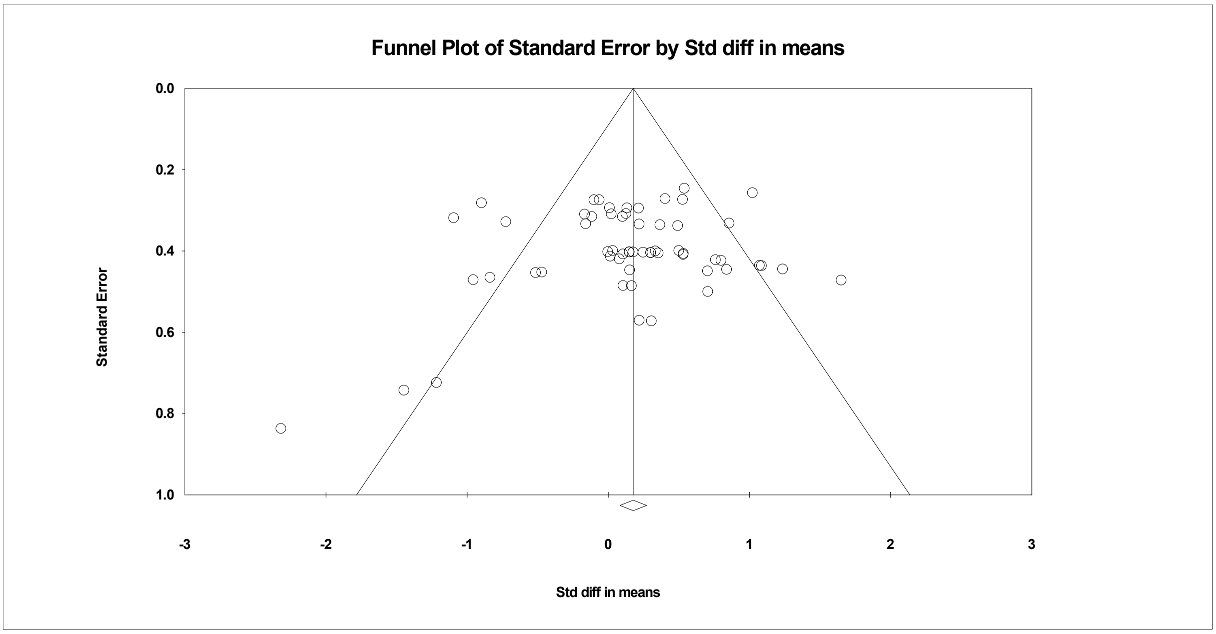

Egger's regression intercept

Intercept	-0.58133
Standard error	0.84806
95% lower limit (2-tailed)	-2.27953
95% upper limit (2-tailed)	1.11688
t-value	0.68548
df	57.00000
P-value (1-tailed)	0.24791
P-value (2-tailed)	0.49582

5. Frail subjects

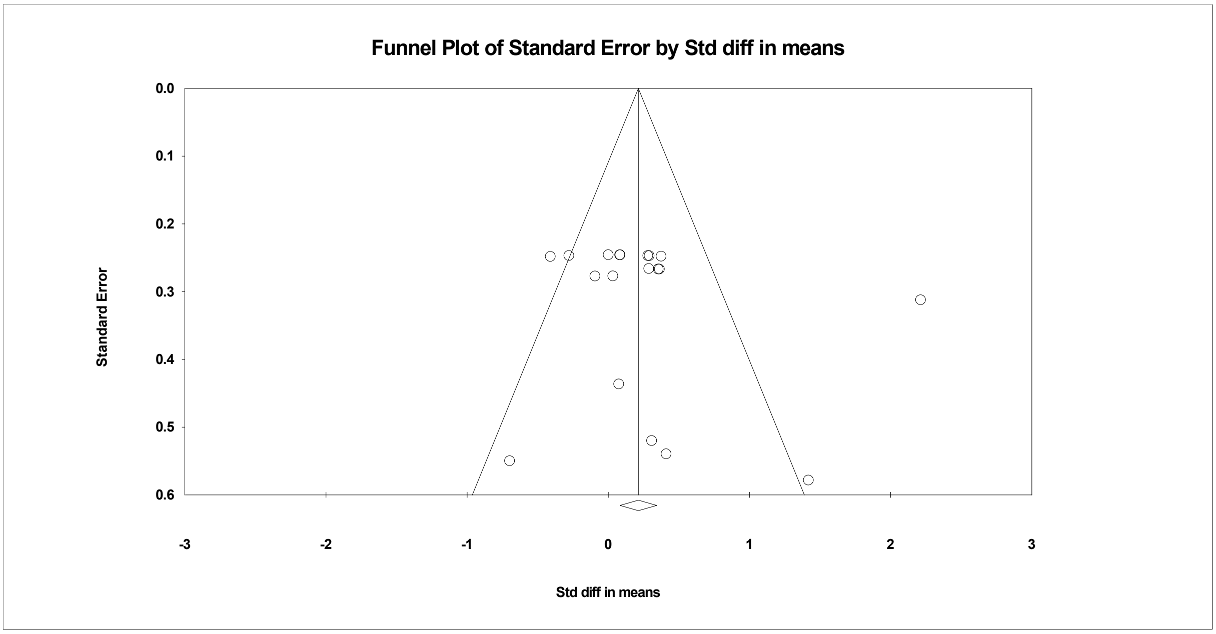

Egger's regression intercept

Intercept	1.37477
Standard error	1.69113
95% lower limit (2-tailed)	-2.19319
95% upper limit (2-tailed)	4.94274
t-value	0.81293
df	17.00000
P-value (1-tailed)	0.21375
P-value (2-tailed)	0.42750
