# Supplementary material for: Effects of digital-based interventions on muscular strength in adults: a systematic review, meta-analysis and meta-regression of randomized controlled trials with quality of evidence assessment
Source: Ann Med. 2023 Jul 15;55(1):2230886. doi: 10.1080/07853890.2023.2230886 (PMC10901531; doi:10.1080/07853890.2023.2230886)
Supplement: Supplemental Material [file IANN_A_2230886_SM1488.pdf]

ID	Year of study publlication	Reference
1	2020	Bostanci, H., Emir, A., Tarakci, D., & Tarakci, E. (2020). Video game-based th
2	2017	Bruno, B., Melissa, V. V., Christophe, B., Sandra, D. B., Serge, V. S. J., & Bart,
3	2017	Calabrò, R. S., Russo, M., Naro, A., De Luca, R., Leo, A., Tomasello, P., ... Bran
4	2014	Cho, H., & Sohng, K. (2014). The effect of a virtual reality exercise program o
5	2021	Cho, H., Song, E., Moon, J., & Hahm, S. (2021). Effects of virtual reality based
6	2012	Daniel, K. (2012). Wii-hab for pre-frail older adults. *Rehabilitation nursing, 3*
7	2016	Eggenberger, P., Wolf, M., Schumann, M., & de Bruin, E. D. (2016). Exergam
8	2015	Eggenberger, P., Theill, N., Holenstein, S., Schumacher, V., & de Bruin, E.D. (
9	2018	Ferraz, D. D., Trippo, K. V., Duarte, G. P., Neto, M. G., Santos, K. O. B., & Filho
10	2020	Feyzioğlu, Ö., Dinçer, S., Akan, A., & Algun, Z. C. (2020). Is Xbox 360 Kinect-b
11	2020	Gallardo-Meza, C., Simon, Kristel., Bustamante, N., Ramirez-Campillo R., Gar
12	2016	Givon, N., Zeiling, G., Weingarden, H., & Rand, D. (2016). Video-games used
13	2015	Gschwind, Y. J., Eichberg, S., Ejupi, A., de Rosario, H., Kroll, M., Marston, H. F
14	2015	Gschwind, Y. J., Schoene, D., Lord, S. R., Ejupi, A., Valenzuela, T., Aal, K., ... D
15	2018	Htut, T. Z. C., Hiengkaew, V., Jalayondeja, C., & Vongsirinavarat, M. (2018). E
16	2013	Jorgensen, M. G., Laessoe, U., Hendriksen, C., Nielsen, O. B. F., & Aagaard, P
17	2013	Kim, J., Son, J., Ko, N., & Yoon, B. (2013). Unsupervised virtual reality-based
18	2019	Kim, K., Choi, B., & Lim, W. (2019). The efficacy of virtual reality assisted vers
19	2016	Kwok, B. C., & Pua, Y. H. (2016). Effects of WiiActive exercises on fear of falli
20	2014	Lee, S. J., & Chun, M. H. (2014). Combination transcranial direct current stim
21	2013	Lee, S., & Shin, S. (2013). Effectiveness of virtual reality using video gaming t
22	2013	Lee, G. (2013). Effects of training using video games on the muscle strength,
23	2015	Lee, M., Son, J., Kim, J., & Yoon, B. (2015). Individualized feedback-based vir
24	2017	Lee, Y., Choi, W., Lee, K., Song, C., & Lee, S. (2017). Virtual reality training wi
25	2016	Lee, S., Kim, Y., & Lee, B. H. (2016). Effect of Virtual Reality-based Bilateral U
26	2019	Liao, Y. Y., Chen, I. H., & Wang, R. Y. (2019). Effects of Kinect-based
27	2015	Liao, Y. Y., Yang, Y. R., Wu, Y. R., & Wang, R. Y. (2015). Virtual reality-based V
28	2020	Lim, D. Y., Hwang, D. M., Cho, K. H., Moon, C. W., & Ahn, S. Y. (2020). *A fully*
29	2020	Lin, R. C., Chiang, S. L., Heitkemper, M. M., Weng, S. M., Lin, C. F., Yang, F. C.
30	2016	Martinho, N. M., Silva, V. R., Marques, J., Carvalho, L. C., Iunes, D. H., & Bote
31	2019	Martin-Martinez, J. P., Villafaina, S., Collado-Mateo, D., Perez-Gomez, J., & G
32	2021	Miclaus, R. S., Roman, N., Henter, R., & Caloian, S. (2021). Lower extremity r
33	2010	Mirelman, A., Patritti, B. L., Bonato, P., & Deutsch, J. E. (2010). Effects of virt
34	2019	Morat, M., Bakker, J., Hammes, V., Morat, T., Giannouli, E., Zijlstra, W., & Do
35	2020	Moreira, N. B., Rodacki, A. L. F., Costa, S. N., Pitta, A., & Bento, P. C. B. (2020
36	2018	Morrison. S., Simmons, R., Colberg, S. R., Parson, H. K., & Vinik, A. I. (2018). S
37	2016	Nagano, Y., Ishida, K., tani, T., Kawasaki, M., & Ikeuchi, M. (2016). Short and
38	2019	Oh, Y. B., Kim, G. W., Han, K. S., Won, Y. H., Park, S. H., Seo, J. H., & Ko, M. H.
39	2018	Park, J., & Chung, Y. (2018). The effects of robot-assisted gait training using
40	2017	Peruzzi, A., Zarbo, I. R., Ceratti, A., Della Croce, U., & Mirelman, A. (2017). Ar
41	2019	Phirom, K., Kamnardsiri, T., & Sungkarat, S. (2019). Beneficial effects of inter
42	2017	Rizzo, J. R., Thai, P., Li, E. J., Tung, T., Hudson, T. E., Herrera, J., & Preeti, R. (2
43	2020	Rutkowski, S., Rutkowska, A., Kiper, P., Jastrzebski, D., Racheniuk, H., Turolla
44	2021	Sadeghi, H., Jehu, D. A., Daneshjoo, A., Shakoor, E., Razeghi, M., Amani, A., H
45	2016	Sajid, S., Dale, W, Mustian, K., Kotwal,A., Heckler, C, Porto, M., ... Mohile, S.(

46	2016	Saposnik, G., Cohen, L. G., Mamdani, M., Pooyania, S., Ploughman, M., Cheu
47	2013	Schoene, D., Lord, S. R., Delbaere, K., Severino, C., Davies, T. A., & Smith, S. T
48	2018	Schumacher, H., Stuwe, S., Kropp, P., Diedrich, D., Freitag, S., Greger, N., ... H
49	2017	Silva, V., Campos, C., Sa, A., Cavadas, M., Pinto, J., Simoes, P., ... Barbosa-Roc
50	2017	Song, J., Paul, S. S., Caetano, M. J. D., Smith, S., Dibble, L. E., Love, R., ... Allen
51	2016	Srikesavan, C. S., Shay, B., & Szturm, T. (2016). Task-oriented training with co
52	2017	Standen, P. J., Threapleton, K., Richardson, A., Connell, L., Brown, D. J., Batte
53	2021	Swinnen, N., Vandenbulcke, M., de Bruin, E. D., Akkerman, R., Stubbs, B., Fir
54	2007	Warburton, D. E. R., Bredin, S. S. D., Horita, L. T. L., Zbogar, D., Scott, J. M., E
55	2020	Yang, C. M., Chen Hsieh, J. S., Chen, Y. C., Yang, S. Y., & Lin, H. C. (2020). Effe
56	2020	Yu, T. C., Chiang, C. H., Wu, P. T., Wu, W. L., & Chu, I. H. (2020). Effects of exe

Country	Type of study	Population assessed		
		Age (mean (	*Gender*	*Health status*
Turkey	RCT	19.8	Mixed	asymptomatic
Belgium	RCT	81	Mixed	hospitalized patients
Italy	RCT	42.5	Mxed	Multiple sclerosis patients
Korea	borderline RCT	59.3	Mixed	hemodialysis patients
Korea	a pilot RCT	69.2	Mixed	Stroke patients
USA	RCT	76.91	Mixed	frailty as defined by Fried et al. (2001)
Switzerland	RCT	74.9	Mixed	Asymptomatic.
Switzerland	RCT	78.86	Mixed	Asymptomatic.
Brazil	Pilot randomized cont	69	Mixed	Idiopathic PD according to the London Brain Bank Criteria
Turkey	prospective, randomi:	50.84	Female	Breast cancer (inclusion criteria: (1) breast cancer surgery (2)
Chile	A block-design randor	68.65	Female	Asymptomatic.
Israel	RCT	59.4	Mixed	individuals with chronic stroke
Germany, Spain a	International, multice	74.7	Mixed	Asymptomatic.
Australia	Permuted block-rand(	80.93	Mixed	Asymptomatic.
Thailand	Single-blind randomiz	75.8	Mixed	Inclusion criteria: 65–85 years, normal cognition, Barthel Inde
Denmark	RCT	74.8	Mixed	Asymptomatic.
South Korea	RCT	68.28	Mixed	Healthy
Korea	a pilot RCT	21.9	Mixed	functional ankle instability
Singapore	A parallel assessor-bli	70.15	Mixed	Asymptomatic.
Korea	a pilot RCT	60.5	Mixed	unilateral hemiparesis caused by stroke
Korea	RCT	74	Mixed	diagnosis of type 2 diabetes
South Korea	RCT	71.71	Mixed	Stroke
Korea	RCT	68.2	Female	Asymptomatic
Korea	RCT	75.93	Mixed	Asymptomatic.
Korea	RCT	71.16	Mixed	hospitalized patients with stroke
Taiwan	RCT	81.8	Mixed	Prefrail and frail elderly
Taiwan	RCT	67.3	Mixed	Parkinson's disease
Korea	a prospective, randon	60.25	Mixed	patients with upper limb dysfunction
Taiwan	RCT	65.7	Mixed	Patients with acute strength
Brazil	RCT	61.5	Female	postmenopausal women
Spain	RCT	53.72	Female	Patients with fibromyalgia
Romania	prospective, randomi:	59.85	Mixed	Stroke patients
USA	a single blind random	62	Mixed	post-stroke
Germany	Three-armed random	69.4	Mixed	Asymptomatic.
Brazil	RCT	70.8	Female	prefrail elderly
England	RCT	66.9	Mixed	Older Adults With Type 2 Diabetes
Japan	RCT	72	Mixed	Asymptomatic
Korea	RCT	55	Mixed	Patients with chronic stroke
Korea	RCT	56.58	Med	Stroke patients
Italy	a single blind random	42.8	Mixed	a diagnosis of relapsing remitting multiple sclerosis
Thailand	A pseudo-randomizec	69.8	Mixed	Asymptomatic.
USA	RCT	56.5	Mixed	Clinical diagnosis of shoulder impingement syndrome
Poland	RCT	61.03	Mixed	Patients with chronic obstructive pulmonary disease
Malaysia	Single-blinded randon	71.8	Men	Asymptomatic.
USA	randomized pilot stud	75	Men	patients with prostate cancer on hormone therapy

Canada	single-blind, parallel-g	62	Mixed	patients with ischemic stroke
Australia	RCT	77.95	Mixed	Asymptomatic.
Germany	prospective randomiz	56.3	Mixed	patients undergoing hematopoietic stem cell transplantation
Portugal	RCT	Not defined	Mixed	Adults diagnosed with Down Syndrome
Australia	Single-blind randomiz	66.5	Mixed	Community-dwelling people with Parkinson’s disease
Canada	RCT	58.2	Mixed	Individuals with rheumatoid arthritis or hand osteoarthritis
UK	RCT	61	Mixed	Patients with a confirmed diagnosis of stroke
Belgium	a pilot RCT	85	Mixed	major neurocognitive disorder
Canada	RCT	22.5	Male	Asymptomatic
Taiwan	RCT	68.12	Mixed	Asymptomatic.
Taiwan	RCT	64	Mixed	Asymptomatic

	Type of control group
Specific population	
aged between 18–24	Passive
Mini Mental Scale Examination (MMSE) > 21/30	Conventional physiotherapy; dual task training
with gait and/or balance problems	robot-assisted gait training
/	Passive
< 50 in Fugl-Meyer assessment	Active (strengthening and stretching exercises)
These characteristics of frailty were unintentional weight loss	Control; seated exercise
/	Balance training group
Residents of retirement homes	Simultaneous cognitive-physical training (tredmill memory wa
regular use of medication for PD	Functional training; bicycle exercise
Department of Multidisciplinary Breast Surgery patients	standardized physiotherapy group
/	Recreationally physical activity sessions
/	Traditional intervention for post stropke patients
community-dwelling older adults	Passive (no training)
/	Educational booklet about evidence-based health and fall pre
Registered in home for the aged	Passive (no training); Physical exercise; Brain exercise
community-dwelling older adults	Passive (no training)
community-dwelling older adults	Passive (no training)
history of two or more ankle sprains in the past 3 months	traditional rehabilitation intervention
of mild-to-moderate physical frailty (9–5 points) on the short	Gym intervention group (cardiovascular training, and balance
first stroke within 1 month prior to enrollment	cathodal Transcranial direct current stimulation during occup
age of > 65 years	Passive
inpatients recovering stroke rehabilitation	Conventional occupational therapy
scores of >23 on the Mini Mental State Examination	postural, balance, functional, lower body coordination, and lo
community-dwelling older adults	Passive (fall prevention education)
Mini-Mental State Examination (MMSE) score of 24–30, Brun	upper extremity training
/	Combined resistance, aerobic, and balance training
Medical centers patients	Contorl (fall-prevention education) & Traditional exercise gro
incomplete motor paralysis due to SCI at the C4–C8 neurologi	conventional occupational therapy
had disability that ranged from minimal (e.g., able to execute	conventional therapy comprised of standardized stroke care a
women aged 50+	pelvic floor muscle training using a gym ball
have been diagnosed with fibromyalgia by a rheumatologist a	Passive (continuing their daily routine)
at least six months post-stroke	Active (standard physiotherapy)
participants with hemiparesis whicih exibit residual gait defici	Active: same exercises but without the feedback
Retired and community-dwelling	Passive (no training)
age > 60	strength, balance, and cardiorespiratory training
/	supervised balance training
Older adults	Passive
Clinically mild to moderate dysfunction of the upper extremit	Conventional occupational therapy
patients who had no overlapping diseases within the past 6 m	Auditory stimulating robot-assisted gait training; general robo
expanded disability status scale EDSS score between 3 and 5.!	Treadmill training
Community-dwelling older adults	Educational material covering cognitive enhancementand fall
subacromial bursitis and/or rotator cuff impingement with po	Conventional SIS therapy protocol
patients with Global Initiative for Chronic Obstructive Lung Di	Traditional pulmonary rehabilitation + exercise capacity traini
Retirement community center	Passive control group and the balance training group
aged > 65	home-based walking and resistance intervention; passive

fi rst-time ischaemic stroke within 3 months of enrolment	Recreational activity
Residents of independent-living units(ILUs)	Passive (asked to continue to perform usual activities)
/	Exercise therapy
/	usual daily activities
/	Passive (no training)
/	finger range of motion exercises and strength exercises
Patienst who still had residual impairment of their arm	Passive (no training)
residents of long-term care facilities	Passive: listening music
engaged in PA below health Canada's recommended threshol	standard bicycle training
/	Conventional exercise group
middle-aged and older adults	Passive

Type of DBI intervention	Strength specific or not
PC-no-exergame	Not (manual and fine motor skills)
PC-exergame	Not
PC-exergame	Not
PC-exergame	Not solely (yoga and strength exercise)
PC-no-exergame	Not (rehab exercises)
PC-exergame	Not (bowling, tennis, and boxing)
PC-exergame	Not (coordination training)
PC-exergame	Not (coordination training)
PC-exergame	Not
PC-exergame	Not (Kinect Sports & Fruit Ninja)
PC-exergame	Not (predominantly balance)
PC-exergame	Not
PC-exergame	Not solely (Balance and strength training)
PC-exergame	Not solely (Balance and strength training)
PC-exergame	Not (upper and lower limb movements & balance training)
PC-exergame	Not solely (Balance and strength training)
PC-exergame	Not (they used Tai Chi and yoga exercises)
PC-exergame	Yes
PC-exergame	Not solely (Balance and strength training)
PC-no-exergame	Not (dexterity)
PC-exergame	Not
PC-exergame	Not (Kinect sports & Kinect adventure)
PC-exergame	Not (balance and coordination training)
PC-exergame	Not solely (coordination, balance and strength)
PC-no-exergame	Not (bilateral upper extremity movements training)
PC-exergame	Not (resistance, aerobic, and balance games)
PC-exergame	Yes
VR-no-exergame	Rehab exercises
PC-exergame	Not solely (training for muscle strength of upper extremities,
PC-exergame	Yes (performing exercises using pelvic movements, maintaini
VR-exergame	Not (postural control, coordination, balance, aerobic conditio
PC-exergame	Not
PC-no-exergame	Not (coordination training)
PC-exergame	Not (coordination training)
PC-exergame	Not solely (strength, balance, and cardiorespiratory)
PC-exergame	Not
PC-exergame	Not
PC-no-exergame	Not (upper-extremity functional ability training)
PC-exergame	Not (coordination training)
PC-exergame	Not (coordination and endurance training)
PC-exergame	Not (coordination training)
PC-exergame	Not (the aim was to restore biomechanical balance across the
PC-exergame	Not solely (agility, dynamic balance, strengthening the lower [illegible]
PC-exergame	Not (coordination, precision and reaction time)
PC-exergame	Not (aerobic and resistance)

PC-exergame	Not solely (flexibility, range of motion, strength, and coordina[illegible]
PC-exergame	Not (coordination training)
PC-exergame	Not
PC-exergame	Not solely (balance, isometric strength exercises and aerobic [illegible]
PC-exergame	Not (coordination training)
PC-no-exergame	Not
PC-no-exergame	Not solely (coordination and strength training)
PC-exergame	Not (coordination training)
PC-exergame	Not (endurance)
PC-no-exergame	Not (Your Shape: Fitness Evolved II). Training static and dynar[illegible]
PC-exergame	Not

Commercial or not	Trained body part
No	Hand movement
Yes (the pirate, wipe out, the flight simulator, hit the box, dro	Whole body
Not	Lower limbs
Yes	Whole body
Yes	Upper body
Yes	Whole body
Yes (Positive Gaming BV, Haarlem, the Netherlands)	Lower limbs
Yes (Positive Gaming BV, Haarlem, the Netherlands)	Lower limbs
Yes	Whole body
	Upper extremity
Yes (sky slalom, sky jump, and balance bubble)	Lower limbs
Yes	Whole body
Not	Lower limbs
Not defined: balance exergames (i.e., walking, stepping, weig	Lower limbs
	Whole body
Yes (balance training:table tilt, slalom ski, perfect 10, tight ro	Whole body
	Whole body
Yes	Lower limbs
Yes	Whole body
Not	Upper body (hands)
Yes	Whole body
	Whole body
Yes	Whole body
Yes	Whole body
Not	Upper limbs
Yes	Whole body
	Lower limbs
Yes	Upper body
Not	Whole body
Yes	Abdominal muscles
Not (designed by the research group, which aims to improve	Whole body
Not	Whole body
No	Lower limbs
Yes (Dividat Senso device (Senso, Dividat, Schindellegi,Switzer	Lower limbs
Yes	Whole body
Yes	Whole body
Not	Lower limbs
Not	Upper limb (while sitting)
Not	Whole body
Not	Lower limbs
Yes	Lower limbs
Yes	Whole body
Yes	Whole body
Yes (The Light Race (Stomp It)mini-game from the Your Shape	Whole body
Yes	Lower limbs

Yes	Whole body
Yes (www.stepmania.com)	Lower limbs
Yes	Whole body
Yes	Whole body
Yes	Lower limbs
Not	Hand dexterity
Not	Upper body
Yes (Dividat Senso device (Senso, Dividat, Schindellegi, Switze	Lower limbs
Yes	Lower limbs
Yes	Core muscles and lower limbs
Yes	Whole body

Outcomes assessed

Which motor ability

Grip strength; pinch strength

Timed chair stands

Hip and knee flexion/extension force

back strength; grip strength; leg strength

Grip strength

30 sec CST; arm curls

5 STS

5 STS (sit to stand 5 times)

sitting raising test

Arm & handgrip strength

5 STS

Grip strength

Knee extensors, hand grip, 5 STS

Knee extensors; 5 STS

Hand grip strength & five times sit to stand

Knee extensors (maximal voluntary contraction; rate force developmer

Muscle strength

plantar flexion, dorsiflexion, eversion and inversion of ankle strength

Knee extensors

Muscle strength

5 STS

Muscle strength

30-sec CST

5 STS

DMMT (he strength of the biceps and triceps); grip test; palmar pinch;

30-sec CST; Grip strength

Hip flexors, hip extensors, knee flexors, knee extensors, ankle dorsiflex

Manual muscle test (elbow flexors, elbow extensors, wrist extensors, fi

Medical research council test: affected side: lower and upper lims; una

pelvic floor muscle assessment

Arm curl

Manual Muscle Test (hip flexion, hip extension, hip abduction, hip add

ankle power generation

Heel rise test; isometric leg extension; leg curl

concentric strength of dominant knee flexors and extensors (peak torq

knee flexion and extension strength

Quadriceps/gluteus medius muscle strength

Manual muscle test (Flexion: shoulder, elbow, wrist, finger / Extension

Medical research council test: Hip flexion, extension, abduction, knee f

Peak hip and ankle generated powers in ST and DT

Knee extensors

Shoulder internal and external rotator strength; scapular stabilizer stre

Arm curl; chair stand

Isokinetic muscle strength of lower limbs (maximum concentric contra

Grip test; chest press maximum repetition test

Grip strength
5 STS; knee extensors
Grip strength
Handgrip test; Standing Broad Jump; 30sec CST; bent arm hang
Average hip abductor peak power; average hip abductor power at low
Grip strength
Grip test
5 STS
Grip strength (combined score of both arms); vertical jump test (dynan
30-sec CST (chair stand test)
Grip strength; 30-sec CST

Type of measurement	*Unit of measurement*
hand-held dynamometer; pinchmeter	kilograms (kg)
Chronometer	Seconds (s)
Lokomat-Pro device, which assesses the muscle isometric for[illegible]	Newton
digital dynamometer	kilograms (kg)
hand-held dynamometer	kilograms (kg)
Paper & Pencil	N of repetitions
Chronometer	Seconds (s)
Chronometer	Seconds (s)
Chronometer	Seconds (s)
Handheld dynamometer	kilograms (kg)
Chronometer	Seconds (s)
hand-held dynamometer; vertec device	kilograms (kg)
Handheld dynamometer	kilograms (kg), seconds (s)
Handheld dynamometer	kilograms (kg), seconds (s)
Handheld dynamometer & 5 repetitions time	kilograms (kg) & time
Static adjustable leg press apparatus	force (N); n of repetitions
MViC dynamometry	force (N)
biodex isokinetic dynamometer	Newton metres (nm)
Handheld dynamometer	% (?)
Manual muscle test (shoulder, elbow and wrist)	MMT grade
Chronometer	Seconds (s)
Manual muscle test	MMT grade
Paper & Pencil	N of repetitions
Chronometer	Seconds (s)
Handheld dynamometer	kilograms (kg)
hand-held dynamometer	kilograms (kg)
Handheld dynamometer	force (N)
Paper & pencil; Handheld dynamometer	graded on a 0 to 5 scale; kilograms (kg)
Paper & Pencil	graded on a 0 to 5 scale
vagynal dynamometry	kilogram/force (Kgf) units
Paper & Pencil	N of repetitions
Paper & pencil	graded on a 0 to 5 scale
force platform	watts per kilogram (W/Kg)
Leg curl machine with force transducers at 200Hz	N of repetitions; Maximal strength (Fmax) and the maximal r[illegible]
hand-held dynamometer	Newton meter per kilogram (Nm/kg); kilograms (kg)
isokinetic dynamometer	kilograms (kg)
hand-held dynamometer; vertec device	kilograms (kg)
Paper & pencil; Handheld dynamometer	graded on a 0 to 5 scale; kilograms (kg)
Paper & Pencil	calculated as 0 points out of a total of 30 points
force platforms (AMTI, 1000 Hz)	Watts per kilo (W/kg)
Handheld dynamometer	kilograms (kg)
a digital scale affixed to a stable platform (a heavy height-adj[illegible]	kilograms (kg)
Paper & Pencil	n of repetitions
Biodex Isokinetic Dynamometer	force (N)
hand-held dynamometer	kilograms (kg)

hand-held dynamometer	kilograms (kg)
Not defined	not defined
hand-held dynamometer	kilograms (kg)
	Paper & pencil; chronometer; hand dynamometer
resistance equipment (Keiser A420, Keiser Sports Health Equi	Watts (W)
hand-held dynamometer; vertec device	kilograms (kg)
Hand dynamometer	kilograms (kg)
Chronometer	Seconds (s)
hand-held dynamometer; vertec device	kilograms (kg); jump height
Paper & Pencil	N of repetitions
hand-held dynamometer	kilograms (kg)

Results	Other comments
Improved hand grip, tip grip and triple grip.	/
No improvement	They also assessed BBS, TUG and 10m DT
Improvements	They also assessed BBS and TUG
Back strength and leg strength improvement	They also assessed balance and flexibility
Improvements in both groups	They also assessed Jebsen-Taylor hand function test (JT) and P
Improvements	They also assessed TUG, Sit and reach and 6MWT
not defined (results are presented, but the p value pre-post is	They also assessed balance, trail making and 4m WT
Improvements	They also assessed gait variables, balance & 3 m-walk test.
Improvements	They also assessed 6MWT, 10MWT and SRT
Improvements in grip but not in pinch	They also assessed ROM, pain intensity & fear of movement
Improvements	They also assessed static balance and the TUG
Improvements	They also assessed gait speed
No improvement in both strength tests; not defined 5 STS	They also assessed TUG, hand reaction time, 10m walking tim
not defined (results are presented, but the p value pre-post is	They also assessed balance, finger press reaction time and TU
Improvement in all measurement	They also assessed balance, cognition, fall concern, exercise e
not defined (results are presented, but the p value pre-post is	They also assessed TUG
Improvements in hip muscle strength (extensor, flexor, adduc	They also assessed ground reaction forces (BALANCE - eyes op
Improvements in plantar flexion	/
Improvements	They also assessed TUG, 6MWD and gait speed
Improvement for shoulder	/
improvement	They also assessed BBS, TUG, FRT, the on-leg-standing test an
Improvements	They also assessed muscle tone & performance of activities-o
improvement	They also assessed the 2-minute step test and the 8-foot up-a
Improvements	They also assessed BBS, FRT and TUG
Improvements	They also assessed upper extremity function using the JHFT, B
Improvements	They also assessed chair sit and reach, 2-minute step, s
Improvement in all measurement	They also assessed sensory integration ability, walking velocit
Improved hand grip for the experimental group	/
improvements	They also assessed the functional status
Improvements	/
Improvements	They also assessed back scratch, sit and reach test and TUG
Exp. g. improved in all parameters; Con. g. not improved in do	They also assessed range of motion
improvement	They also assessed gait speed
Improvements in heel rise test but not the Maximal leg extens	They also assessed reactive balance, functional balance & TUG
Improvements in peak torque knee extensors and 5 STS	They also assessed TUG, gait speed, and balance
No improvement	They also assessed simple reaction time, lower limb proprioce
Improvements	They also asssessed TUG, 10MWT, tandem gait test, one-leg-s
Improvements	They also assessed Box and Block Test and 9-Hole Peg Test (9
Improvements	They also assessed BBS, TUG, 10MWT and the Fugl-Myer asse
Improvements	They also assessed 6MWT, 10MWT, TUG and FSST
No improvement	They also assessed fall risk (Physiological Profile Assessment a
No improvement	They also assessed ROM analysis
Improvements	They also assessed back scratch, chair sit and reach, Up and G
Peak torque of quadriceps and hamstrings for the dominant a	They also assessed single-leg stance test, tandem stance test,
No improvement	They also assessed 6MWT

to define	/
not defined (results are presented, but the p value pre-post is	They also assessed choice stepping reaction time, TUG, postur
No improvement (the strength sig. decreased - as right after t	They also assessed the 2 MWT
Improvements in the handgrip test, standing broad jump	They also assessed plate tapping, shuttle run, flamingo balanc
No improvements	They also assessed stepping performance, reaction time, mob
to define	/
Improvement at the 2nd assessment (midpoint)	They also assessed Wolf Motor Function test, Nine-Hole Peg a
not defined as part of the SPPB	They also assessed gait speed, standing balance test, step rea
Improvement in vertical jump test	They also assessed flexibility and maximal aerobic power
Improvements	They also assessed TUG, FRT and OLST (one-leg stance test) w
Improved the 30-sec CST	They also assessed flexibility, balance and 6MWT
